# ‘Killing Me Softly With His/Her Song’: How Leaders Dismantle Followers’ Sense of Work Meaningfulness

**DOI:** 10.3389/fpsyg.2018.00654

**Published:** 2018-05-09

**Authors:** Petra Kipfelsberger, Ronit Kark

**Affiliations:** ^1^Institute for Leadership and Human Resource Management, University of St. Gallen, St. Gallen, Switzerland; ^2^Department of Psychology, Bar-Ilan University, Ramat Gan, Israel

**Keywords:** work meaningfulness, meaning making, leadership, followership, dark triad

## Abstract

Leaders influence followers’ meaning and play a key role in shaping their employees’ experience of work meaningfulness. While the dominant perspective in theory and in empirical work focuses on the positive influence of leaders on followers’ work meaningfulness, our conceptual model explores conditions in which leaders may harm followers’ sense of meaning. We introduce six types of conditions: leaders’ personality traits, leaders’ behaviors, the relationship between leader and follower, followers’ attributions, followers’ characteristics, and job design under which leaders’ meaning making efforts might harm or ‘kill’ followers’ sense of work meaningfulness. Accordingly, we explore how these conditions may interact with leaders’ meaning making efforts to lower levels of followers’ sense of meaning, and in turn, lead to negative personal outcomes (cynicism, lower well-being, and disengagement), as well as negative organizational outcomes (corrosive organizational energy, higher turnover rates, and lower organizational productivity). By doing so, our research extends the current literature, enabling a more comprehensive understanding of leaders’ influence on followers’ work meaningfulness, while considering the dark side of meaning making.

## Introduction

The dominant perspective on leaders’ meaning making role, in both the leadership and the meaningfulness literature, focuses on leaders’ positive influence on followers’ work meaningfulness. This line of research has shown that transformational leadership, empowering leadership, and high-quality leader-member relationships are positively related to followers’ perceptions of work meaningfulness (e.g., Piccolo and Colquitt, 2006; Arnold et al., 2007; Grant, 2012; Tummers and Knies, 2013). However, research on the dark side of leaders’ meaning making is scarce (for exceptions, see Amabile and Kramer, 2012; Neal et al., 2013; Bailey and Madden, 2016). While prior research has already shown that not all leaders’ efforts to infuse the work of followers with positive meaning are effective (e.g., Kirkpatrick and Locke, 1996), we know very little regarding whether and under which conditions leaders’ attempts to instill work meaningfulness among followers might have detrimental effects on followers’ work meaningfulness. Investigating the potentially harmful effects of leaders’ meaning making is a pressing endeavor since the quest for meaningful work among many employees is increasing (Cascio, 2003). People across generations and particularly today’s emerging adults [Millennials, born after 1980, also described as “generation me” by Twenge (2006)] are motivated to realize their selves at work and focus on having work opportunities that will enhance their personal sense of meaningfulness. However, at the same time, many people lack a deeper sense of meaning at work and are searching for something that is larger than themselves and that goes beyond their ego (Lancaster and Stillman, 2010; Lepisto and Pratt, 2017). Organizations might react to the increasing need for meaningful work by intensifying leaders’ meaning making efforts (Cascio, 1995; Feintzeig, 2015). Therefore, it is important to better understand how and under which conditions intense efforts of leaders’ meaning making may backfire and decrease work meaningfulness among followers.

Our conceptual model introduces six types of conditions under which leaders’ meaning making efforts might harm followers’ meaningfulness at work: leaders’ personality traits, leader behaviors, the relationship between leaders and followers, followers’ attributions toward their leaders, followers’ characteristics, and job design. This model makes important contributions to the literature on meaning making, work meaningfulness, and leadership in the following ways. First, our research provides a conceptualization, grounded in theory and empirical findings, of several conditions under which leaders’ meaning making may have detrimental effects on followers’ work meaningfulness. In such, we provide a novel perspective on the influence of leadership on meaningfulness (e.g., Shamir et al., 1993; Podolny et al., 2005). The conceptual model we develop contributes to the growing literature on the ‘dark side of leadership’ (e.g., Mathieu et al., 2014) and destructive leadership (e.g., Einarsen et al., 2007). Second, by incorporating the recent advancements of research on work meaningfulness (Martela and Steger, 2016; Lepisto and Pratt, 2017), we develop a more detailed understanding regarding the particular dimensions of work meaningfulness (coherence, purpose, or significance), as well as of the pathways (realization or justification perspective) that can be harmed by the interaction of leaders’ meaning making and the identified conditions. Third, through revealing the factors under which leaders’ meaning making might harm followers’ work meaningfulness, we extend prior writings on the critical perspective on the management of meaning (Lips-Wiersma and Morris, 2009; Bailey et al., 2017). Finally, we present an agenda for future research and discuss practical implications of our conceptualization.

### Harming Followers’ Sense of Meaningfulness

Studies on the cultivation of work meaningfulness have mostly focused either on work conditions (e.g., May et al., 2004; Humphrey et al., 2007), on individuals’ personal behaviors (e.g., Vuori et al., 2012) that increase their sense of meaningfulness, or on a combination of both (e.g., Chalofsky and Krishna, 2009) as antecedents to work meaningfulness. Studies that have addressed leadership as an antecedent, have generally focused on how leaders help construct work meaningfulness among followers and contribute to their sense of meaning (e.g., Shamir et al., 1993; Zhang and Bartol, 2010; Tummers and Knies, 2013). There are different views of leaders’ role in meaning making and how meaning making leads to meaningful work. While the top-down views (e.g., job design, leadership style and behaviors; Piccolo and Colquitt, 2006; Grant, 2012) consider leaders as the agents in meaningful work, the bottom-up view (e.g., job crafting; Wrzesniewski and Dutton, 2001) treats employees as the agents that construct their own sense of meaning at work. We integrate both perspectives (top–down and bottom–up; leaders as well as followers as agents) in our model. We transfer recent research advancements on meaning in life to the context of meaningful work and suggest that meaningful work consists of three components: *coherence*, which refers to employees’ understanding and ability to make sense of what is happening at work; *purpose*, which refers to directionality of employees’ work and the ability to connect their work to a higher-order goal; and *significance*, which refers to employees’ evaluation of their work worth (Martela and Steger, 2016).

Harming followers’ work meaningfulness refers to situations in which any of the three components- *coherence*, *purpose* or *significance-* of followers’ work is diminished. First, regarding coherence, this implies that followers struggle to understand the meaning of their work; they find their work chaotic, unstructured, do not know or lose track of what their work is all about and are unable to grasp the point of their work *(lack of coherence)*. Second, a reduction of followers’ purpose means that the experience, belief, or hope of followers that their work makes a positive difference in the world is reduced, and that they do not see a clear direction and contribution of their work. They might feel that their work is going nowhere or that their work does not serve a higher-order goal *(lack of purpose)*. Third, regarding significance, the reduction of feelings of one’s work significance refers to the worth, value, and importance of one’s work. Followers might experience that their work is useless and not worthwhile. They might struggle to explain the worth of their work, be unable to justify the worth of their work, or consider the worth of their work and tasks as ambiguous. Furthermore, they may lack solid accounts for the worth of their work, or their established accounts, based on personal values and what matters for them, might have been impaired (*lack of significance*; Martela and Steger, 2016; Lepisto and Pratt, 2017).

While these three components of work meaningfulness are the elements that might be dismantled by leaders’ meaning making, we draw on Lepisto and Pratt’s (2017) recent work to explain what drives and underlies the process of meaning erosion. These scholars differentiate between two ideal-type conceptualizations of meaningful work in the vast literature: the *realization and justification* perspective (Lepisto and Pratt, 2017). We propose that this dual perspective on meaningful work helps shed light upon the deeper, underlying mechanisms that can be harmed by leaders’ meaning making. The realization perspective of work meaningfulness refers to the idea that individuals strive to express and realize themselves through their work. According to this perspective, meaningfulness is achieved through fulfillment of motivations, desires, and needs associated with self-actualization (Lepisto and Pratt, 2017). For example, leaders that have a strong ideological base and are obsessed with their own frame(s), voice or “song” of meaning, can offer a frame of meaningfulness that will override employees’ inner and authentic sense of meaningfulness. This can hinder employees’ ability to *realize* and take ownership over their own sense of work meaning. The justification perspective, on the other hand, refers to an individual’s ability to account for one’s work worth and to consider one’s work as worthy (Lepisto and Pratt, 2017). For example, leaders that use incoherent accounts, suggest unethical purposes, or rely on unethical means to mobilize followers, can distort followers’ foundation for *justifying* the worth of their work toward others or for considering their work worthwhile for themselves.

**FIGURE 1:**
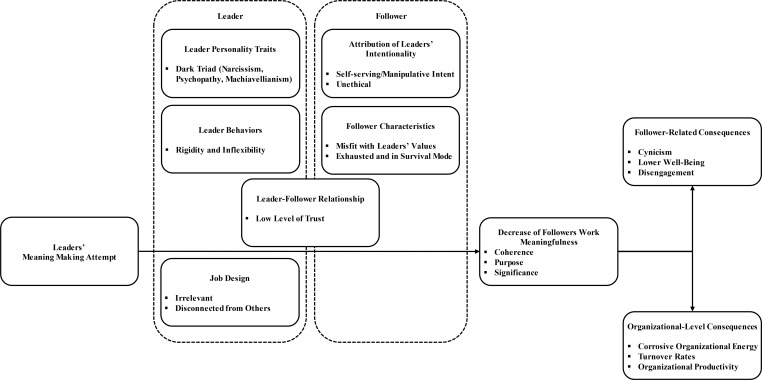
Conceptual model.

In the following sections, we elaborate in depth upon each of the circumstances that we propose might harm followers’ sense of meaning at work, discuss the particular component of meaningful work that might be negatively affected and refer to the underlying mechanism of realization and/or justification. We provide propositions for each condition. **Figure 1** depicts the conceptual model.

## Components of the Conceptual Model

### Leaders’ Personality Traits: The Dark Triad

We begin with leaders’ personality traits and focus specifically on the dark triad because we expect a particularly large negative effect of these traits on followers’ meaningfulness. All three personality dimensions– namely, psychopathy, narcissism, and Machiavellianism – entail a socially malevolent and rather insincere character and the behavioral tendencies to promote oneself, while interacting with others in an aggressive and emotionally cold way (Paulhus and Williams, 2002). More specifically, psychopaths lack a conscience, and are therefore not bothered by and do not try to change their bad and hurtful behavior (Tamayo and Raymond, 1977; Babiak and Hare, 2006). In fact, they have been shown to get an exciting thrill from hurting others (Clarke, 2009). They also lack emotions (Nadis, 1995; Stout, 2005), do not consider others’ pain when making moral judgments (Blair et al., 1995), and are not sensitive to criticism. Narcissists are primarily driven by self-enhancement and need constant external self-affirmation. Although they do not intend to harm others, they ignore others’ welfare (Braun, 2017). Machiavellians, though they do not lack a conscience, they do lack concern for conventional morality, ignore interpersonal affect, have low ideological commitment, and are quite adapt at manipulating others and are willing to do so through all means (McHoskey et al., 1998).

Though psychopathy, narcissism, and Machiavellianism are distinct constructs, all are characterized by a high degree of selfishness and a willingness to put one’s own needs ahead of others (O’Boyle et al., 2012). We argue that the combination of leaders’ meaning making and any one of the dark triad traits might reduce followers’ work meaningfulness due to the overly self-focused and overly socially dominant characteristics of such leaders (Rosenthal and Pittinsky, 2006), which might overshadow followers’ ability to develop or follow their own inner sense of meaning. In fact, such self-focused personalities might expect unquestioning obedience (O’Connor et al., 1996) as well as full identification and compliance with their sense of meaning from their followers (Jones et al., 2004; Vaknin, 2009). Their strong attachment to their self-promoting motives is very likely to hamper or dismantle employees’ inner sense of meaning. Besides their desire for compliance, these personalities are also associated with a lack of empathy, which is the inability to understand and share the feelings of others. Their lack of empathy makes it difficult or impossible to promote the meaning making of their followers because they do not know and value the needs, desires, and feelings of their employees.

Beyond the theoretical links between dark triadic leaders’ characteristics and their effects on followers’ work meaning, such leaders’ behaviors might affect and interfere with the various components of work meaningfulness (coherence, purpose, and significance). In terms of *coherence*, there is evidence that corporate psychopaths engage in extreme forms of mismanagement, characterized by poor personnel management, directionless leadership, and mismanagement of resources (Babiak and Hare, 2006; Boddy et al., 2015). In addition, employees working under corporate psychopaths receive less instruction, less training, less help and they experience more unfairness from their supervisors (Boddy, 2010). This sort of chaotic and precarious managing style is antithetical to clarity, and to the ability to make sense of one’s work and work environment and can therefore directly obstruct employees’ sense of coherence.

In terms of *purpose*, empirical studies of corporate psychopaths have shown that they create a toxic work environment, characterized by conflict, bullying, increased workload, low levels of job satisfaction, and unnecessary organizational constraints (Babiak and Hare, 2006; Boddy, 2010). Moreover, dark triad leaders are motivated by personal gain and self-promotion (Jakobwitz and Egan, 2006; MacNeil and Holden, 2006) rather than that of their organization, employees, or other stakeholders (see Boddy, 2006 on corporate psychopaths; Holian, 2006 on narcissists) or greater society. For example, a prior study suggests that there is a negative relationship between corporate psychopaths in organizations and employees’ perceptions of corporate social responsibility (Boddy et al., 2010). Such lack of a higher-order prosocial goal might reduce followers’ feelings of purpose. Moreover, employees of managers possessing dark triad traits may come to dread their work due to adverse experiences at their workplace, leading them to overlook any purpose their work might serve. Indeed, all three traits have been associated with bullying behavior (Baughman et al., 2012), with the use of manipulation tactics (Jonason et al., 2012), and have been linked to counterproductive work behavior (O’Boyle et al., 2012). Therefore, it is reasonable to assume that managers possessing any one of these traits create unpleasant work environments for their employees, void of safety, integrity, and pleasantness, which may overshadow employees’ ability to view their work positively and as serving a higher purpose.

In terms of *significance*, there is evidence that leaders with dark triad traits do not provide followers with grounds for feeling that their work is appreciated, valued and important but rather cause them to feel quite the opposite. Research has shown that employees were significantly less likely to feel that they receive recognition for doing a good job, that their work is appreciated, and that they were properly rewarded for their efforts, when corporate psychopaths hold leadership positions (Boddy et al., 2010). The dominance and obsession by the self-serving motives of such leaders might degrade followers as human beings, their ideas, and their contributions to the collective. Research has shown that leaders’ narcissism lowers employees’ self-esteem and, in turn, their level of creativity (Eissa et al., 2017). Hence, we argue that followers who are treated in an instrumental manner and used to serve the leader’s interest, might not only feel deliberately misused and devalued but also may feel disenabled to realize their self or their interests at work and therefore their sense of significance at work will be lowered.

Proposition 1: Leaders’ dark triad personality traits (narcissism, psychopathy, and Machiavellianism) will moderate the effect leaders’ meaning making has on followers’ work meaningfulness, such that this relationship is reduced if the leader scores high on any of the dark triad traits.

### Leaders’ Behavior: Rigidity and Inflexibility

There are multiple leadership behaviors that can reduce followers’ sense of meaning. Here, as a central example, we focus on behaviors that highlight leaders’ rigid and inflexible behaviors. Several facets of inflexibility and rigidity that pertain to leadership are introduced in the organizational literature (Good and Sharma, 2010). In our conceptual model, we discuss two specific facets that we estimate to moderate the effects of leaders’ meaning making attempts on followers’ sense of meaning. We argue that leaders with *low cognitive flexibility* and *low communication flexibility* – accumulating in rigid behaviors of leaders – can hurt followers’ sense of work meaningfulness, through leaders’ inability and unwillingness to adjust to and incorporate new situations (those pertaining to followers, in particular).

Cognitive flexibility is defined as the ability to shift attention in order to respond to the environment in a new way (Good and Sharma, 2010). Leaders low on cognitive flexibility struggle to overcome their fixed mental schemas and are unable to come up with situation appropriate responses. Cognitive inflexibility is therefore closely related to rigidity, which has been defined as “the inability to produce novel or changed responses” (Vacchiano et al., 1969, p. 268). Consequently, such leaders are “closed-minded,” inflexible, rigid, and do not adjust their behavior according to situational demands but rather adhere to their way of seeing the world and acting upon it.

Another aspect of flexibility that can affect followers’ sense of meaning is communication flexibility, which has been defined as the ability to generate and select communicative options according to the needs of the situation (Martin and Rubin, 1994). This form of flexibility has been proposed to be important for communicating continuously shifting goals and expectations in the dynamic environments in which leaders operate. In other words, leaders are often required to change their verbal and non-verbal communicative behaviors according to the situation and audience (Stevens and Campion, 1994). Indeed, studies show that leaders’ choice of words, symbols, and expressions influence the degree to which the audience becomes inspired, aroused, and committed (Awamleh and Gardner, 1999).

Taken together, if leaders are low on cognitive flexibility—thus, unable to shift the work goals and tasks according to the situational demands— and low on communication flexibility —thus, unable to communicate clear expectations, goals, and tasks— they may weaken followers’ sense of coherence, purpose, and significance in the following ways. Regarding followers’ coherence, if employees do not understand what the leader is trying to convey due to leaders’ very limited range of words and phrases, overly abstract or overly detailed speech (low communication flexibility), followers’ ability to understand and make sense of their work will be compromised. In addition, if the leader has low cognitive flexibility, the leader may not be able to change the meaning they provide for work according to their audience. In these situations, the leader is more likely to distort followers’ ways of realizing their selves at work and their ways of justifying the worth of their work rather than facilitate these. Followers are likely to be left with an unstructured, chaotic picture of their work and might struggle to make sense of their work based on leaders’ thoughts and words.

Even worse, regarding followers’ work significance, cognitively inflexible leaders might be so rigid in their opinions and evaluations that they squelch followers’ sense of meaningfulness of and at their work as they provide too little space for followers to self-realize and to express themselves. Metaphorically speaking, ‘his/her personal song is so loud,’ pervasive, and insistent that followers’ ‘song’ is not heard or that followers stop singing. In addition, lack of cognitive flexibility implies an inability to engage in perspective taking. Lacking the ability to see things from the other’s point of view and to integrate another’s perspective, makes it difficult for a leader to tap into the values of their followers. In addition, as such leaders overlook what is important to their followers and only emphasize their own view and values, followers’ feelings about their self-worth and the worth of their work are likely to be reduced.

Taking these characteristics one step further and considering the dimension of purpose, cognitive and behavioral rigidity is a characteristic of the ideological or dogmatic leader who holds strong personal convictions and values that mostly refer to the past: “The ideological leader, moreover, will justify actions based on a limited number of relatively inflexible core beliefs and values. Appeal to others will be based, not on the leader per se, but rather the truth embedded in these beliefs and values” (Mumford and Strange, 2013, p. 133). In addition, ideological leaders have been described as extremely focused on the past, which they oftentimes idealize (Mumford, 2006), so that their mental models are not constructed around future goals but rather upon goals that have served them well in the past (Mumford et al., 2008). While followers might be looking for something valuable to contribute to in the future and might wish to pursue different goals than their leaders, who glorify the past and their personal (at times limited) thinking, followers’ sense of purpose is likely to be diminished by leaders’ rigid ideological past-oriented convictions.

Overall, the inflexible and rigid thinking and behaviors of leaders is likely to hurt followers’ potential for self-realization at work. Followers may be provided with too little space to discover and express their true selves at work, be intimidated and miniaturized when leaders are so small-minded, fixated, and focused on their personal truth and convictions. Besides hurting their self-realization, followers’ justification base might be diminished if only leaders’ thoughts and words are allowed to be used to justify the worth of their own work. The narrow-mindedness of leaders might also limit followers’ ability to think more openly and flexibly in the long-term hurting the likelihood of them finding salient accounts for the worth of their work on their own in the long-term.

Proposition 2: Leaders’ behavior will moderate the effect of leaders’ meaning making on followers’ sense of meaning such that followers’ sense of meaning will be lowest, if leaders behave in rigid ways (low cognitive and communication flexibility and strong adherence to their own ideological convictions).

### Leader–Follower Relationship: Low Level of Trust

The relationship between leaders and followers is likely to affect the ways in which followers make sense of how leaders interpret situations and craft meaningfulness. One of the central elements of a relationship is the level of trust among leaders and their followers (Barnard, 1938). We suggest that leaders’ meaning making might reduce followers’ sense of work meaningfulness if followers have a low level of trust in their leaders. Followers’ trust in their leaders depends on their perceptions of leaders’ ability, integrity, and benevolence (Mayer et al., 1995). Leaders’ *ability* refers to their capabilities, competence, experience, skills, and qualifications and to their professional behavior related to their role as leaders (Lapidot et al., 2007). Perception of leaders’ *integrity* refers to followers’ perceptions that their leader sticks to a set of values and principles, that are acceptable to them (Mayer et al., 1995) and includes leaders’ behavior that displays honesty, loyalty, and taking responsibility (Lapidot et al., 2007). Perceptions of leaders’ *benevolence* refers to followers’ belief that their leader has good intentions to contribute to followers’ wellbeing (Mayer et al., 1995) including support, caring, and encouragement (Lapidot et al., 2007).

If leaders are seen by followers as low on their ability to perform their role and lead the organization to reach its expectations, followers’ sense of meaningfulness might decrease for several reasons. First, if leaders are unable to effectively coordinate and orchestrate followers’ activities, followers’ work coherence is likely to be compromised. If a leader’s ability is lacking to the extent that s/he cannot effectively direct employees toward completing tasks and reaching goals, then followers’ sense that their work has the potential to make a meaningful contribution and hence, its worth, are likely to be reduced. Second, when leaders are unable to effectively communicate, instruct and manage complexity, followers’ work coherence, and, in turn, significance, are likely to be harmed. If followers do not comprehend the meaning of their work and find coherence, they are quite unlikely to consider their work as important and significant (Martela and Steger, 2016). Third, leaders’ ability to make decisions and their comfort in and after doing so might also have crucial influence on followers’ sense of meaning, based on cross-cultural research on the differentiating factors of leaders’ effectiveness (Brodbeck et al., 2000). If leaders are indecisive, feel uncomfortable and unconfident in making decisions (Brodbeck et al., 2000), followers might not only struggle to trust leaders’ decisions but also might not see a clear course of action, worry about wasted efforts and resources, so that their sense of coherence and purpose is threatened and reduced. Research has indicated that leaders’ indecisiveness might evoke employees’ anxieties of being ‘adrift in the ocean’ (Mulki et al., 2012), which might be directly linked with followers’ lower work coherence and, hence, indirectly also hurt their feeling of purpose. If leaders do not make decisions, or if they tend to regret their decisions immediately after making them, or take them back frequently (Amabile and Kramer, 2012), followers might have a hard time understanding how their work activities fit together (coherence) and how they might contribute to the broader picture (purpose), as their leader takes no clear course of action.

Concerning integrity, the second major aspect of trust, followers may not believe the leaders’ construction and framing of the meaning of their mutual work, if they perceive their leader as being low on integrity. If leaders do not serve as role models, do not ‘practice what they preach,’ do not keep their promises and do not tell the truth (Cha and Edmondson, 2006), followers might question leaders’ meaning making attempts, and find it difficult to embrace their messages regarding the purpose and significance of their work. Leaders’ inconsistencies, such as changing their messages too often or provision of contradicting messages, not only thwart work coherence, but also reduce followers’ perceptions of leaders’ credibility, including that of the messages they provide regarding work significance and purpose, and thus impair followers’ belief in their work’s meaningfulness.

Regarding benevolence, the third component of trust, when followers question leaders’ benevolence, meaning they do not believe their leader has their best interest at heart, they might be inclined to reject the leader’s attempts to tap into their self-realization goals and justifications, as they may view such attempts as insincere acts brought on by ulterior motives. Worse, if they think their work serves a malevolent agenda of their leader, they may view their work as such that yields negative consequences, which is likely to negatively affect their perspective regarding their work as positive and significant.

In addition to a low level of trust in one’s own leader, a general distrust toward all leaders, based on previous personal experience with other leaders (i.e., role-based trust, Kramer, 1999) or bias stemming from a perceived prevalence of managerial misconduct (often reported by the media; Greve et al., 2010), might also negatively influence the degree to which followers trust their leaders, including what these leaders say, preach, or do. Thus, we propose that:

Proposition 3: Followers’ trust in their leaders will moderate the effect of leaders’ meaning making on followers’ sense of meaning such that followers’ sense of meaning will be reduced the lower their trust in their leaders is (e.g., lowest if they lack trust in their leaders).

### Followers’ Attributions Toward Leaders’ Intentions

Above, we focused on the characteristics of leaders, their behaviors, and their relationships with followers. While these actual characteristics interact with leaders’ ability to foster or harm a sense of meaning among followers, another key aspect, which needs to be taken into consideration, is what followers ascribe and attribute to their leaders. A major stream in the leadership literature highlights the crucial role of followers’ attributions toward their leaders in shaping how leaders’ behaviors and intentions are understood and evaluated by followers, as well as how they affect the leader-follower interaction (e.g., Ferris et al., 1995; Martinko et al., 2007). For example, the romance of leadership phenomenon, suggested by Meindl et al. (1985), captures a phenomenon showing that extreme performance outcomes (very poor or very high outcomes) is often attributed to the effects of leadership, although in fact the resulting performance might be due to the situation, such as market development. Furthermore, extant research showed that the way followers perceive the intentionality of leaders’ behaviors influenced their interpretations of leaders’ actions as well as the followers’ reactions (Ferris et al., 1995). Although leaders might have good intentions when they try to infuse meaningfulness into the workplace, the effects of their attempts interact with the attributions of followers, namely how they understand and interpret this behavior. In the following, we focus on the situations, in which followers perceive leaders’ intentions as self-serving and manipulative or as unethical, because both might negatively affect followers’ subsequent work meaningfulness.

If followers attribute leaders’ meaning making to leaders’ self-serving motives, their sense of meaningfulness might be distorted. Leaders, who are perceived as aiming to influence followers for their own self-interests, such as solely increasing their own power, prestige, or advancing their own career, might reduce followers’ ability and willingness to relate their own work activities to a higher-order purpose. Feelings of ‘I am being used’ (Dienesch and Liden, 1986) might arise among followers and lower their self-esteem, self-confidence and, importantly, their ability to realize their selves at work. Even if followers enjoyed their work initially, the feeling that their talents, efforts, and potentials are being used for leaders’ self-serving purposes might harm their willingness to fully engage in their work and undermine their answer to the question “does my work reflect and fulfill who I am” (Lepisto and Pratt, 2017, p. 111). Furthermore, followers are likely to experience anger and disappointment if their sole answer to the question of “why their work is worthy” is because it helps their leader get ahead or gain more benefits (salary, prestige, etc.). This is because people across different cultures and professions are motivated by the need to make a difference to others (Schwartz and Bardi, 2001), which likely does not mean serving leaders’ egocentric or profane interests. While leaders might vary in the degree to which they are able to hide their self-serving motives, followers’ work meaningfulness might be even more negatively impacted if leaders try to manipulate followers by their meaning making attempts. Leaders’ manipulation has been shown to hurt the leader-follower relationship and followers’ outcomes (Lin et al., 2016). Although the leader might have collective intentions in mind and refer to collective motives (e.g., a higher-order purpose), their meaning making behavior might backfire if followers attribute hypocrisy to it. A hypocritical leader violates and only pretends to care about the company’s values but in fact has self-serving intentions since hypocrisy is a deliberate violation of (espoused) values (House and Howell, 1992; Cha and Edmondson, 2006).

Another possible attribution of followers toward leader behaviors refers to unethical motives, that is, to intentions that are morally unacceptable to the larger community (Jones, 1991) and that violate moral norms (cf. Kaptein, 2008). If leaders make meaning and are perceived by followers as such that rely on unethical motives, followers might experience a moral dilemma because they are supposed to support their leader and to contribute to the leader’s success through their work, while at the same time they experience that their leader represents and/or engages in unethical issues. The unethicality of leaders might particularly hurt followers’ base of justifying the worth of their work and their experience of their own work’s worth. If followers feel that part or all their work activities violate morale standards or norms, they might have a hard time viewing their work as significant and positive. The experience of meaningfulness is socially constructed and determined (Wrzesniewski et al., 2003; Lepisto and Pratt, 2017). Hence, if leaders’ meaning making is altogether or partly morally inacceptable to the larger community, the accounts that the leader provides, might also be morally unacceptable for their followers and prevent them from developing and maintaining a positive work identity. Moreover, followers’ attributions of leaders’ motives as unethical might also diminish their ability and willingness to realize their (full) selves at work. The violation of moral standards might motivate followers to (emotionally) distance themselves from their leader and/or their work instead of unleashing their potential to fully engage in their work. Such distancing – necessary for followers to not blindly comply with leaders’ unethicality – is likely to reduce their level of purpose and significance.

Proposition 4: Followers’ attributions toward leaders’ meaning making attempts will affect the way they interpret and internalize a sense of work meaningfulness, such that when followers perceive leaders a) as manipulative and self-serving, or b) unethical, leaders’ conveyed sense of meaningfulness will lead to lower levels of followers’ work meaningfulness.

### Followers’ Characteristics

We also consider the characteristics of followers as conditions that might influence the effects of leaders’ meaning making attempts on followers’ sense of meaning. In particular, we focus on followers’ personal values and their extent of misfit with leaders’ values as well as on followers’ emotional state. We argue that the combination of leaders’ meaning making and the misfit between leaders’ and followers’ values will reduce followers’ level of meaningfulness because the values prescribed by leaders as important might not matter or might be less meaningful to followers and hence, will reduce the level of their work significance. Researchers studying incidents in which followers lost their sense of meaningfulness discovered that employees related to events of leaders’ behavior that were disconnecting them from their own set of values (Bailey and Madden, 2016). If followers value something different than what their leader values, their ability to realize their self through work and what matters to them are distorted. Feelings of self-alienation among followers whose values are in conflict with leaders’ meaning making might increase. Notably, the extent of value misfit among leaders and followers might play an important role. While a small incongruence could even be beneficial in order to expand followers’ perspective and to transcend their own values and interests, which is a fundamental aspect of transformational leadership (Sosik, 2005; Brown and Treviño, 2009), a fundamental conflict between their values and the values the leader promotes – be it the leader’s own personal values or organizational values – might undermine the experience of followers’ work meaningfulness.

Besides the threat to one’s self-realization, the justification of meaning might also suffer in case of a value misfit because followers need anchors, such as values, to competently justify the worth of their work (Sennett, 2006; Lepisto and Pratt, 2017). If the leader offers accounts for the worth of one’s work, but the follower does not buy into due to the value incongruence or value conflict, the individual’s sense of coherence and purpose might be crumbled. The reason for the reduction of coherence is that followers, who have a basic or solid understanding of how their work activities yield a holistic entity, might be irritated by a different perspective that is constantly suggested by the leader that is incongruent with their personal values. Furthermore, followers’ ability to experience a higher-order purpose might also be harmed if the leader preaches a higher-order purpose (based on personal and/or organizational values) that fundamentally conflicts with the followers’ values. Followers, who are unable to internalize the higher-order purpose proposed by the leader, due to the value conflict, might struggle to understand the worth of their work. Together, both perspectives of realization and justification suggest that followers’ misfit with leaders’ values is a factor that is likely to yield negative effects for leaders’ meaning making on followers.

While the misfit with leaders’ values is deeply rooted in followers’ more stable personal characteristics, there may also be emotional components of followers, which are likely to interact with leaders’ meaning making processes. For example, if followers are emotionally exhausted by their work due to overwhelming intense situations (e.g., conflicts with customers; Schaufeli and Bakker, 2004), they might enter survival operational mode; meaning, they may try to solve immediate problems and to get things done (Kahn et al., 1964; Edwards, 1992). Accordingly, we argue that this type of followers’ mode might clash with leaders’ meaning making attempts so that it reduces followers’ sense of meaning.

We assume that leaders’ meaning making entails an idealized, promotion-oriented focus. The leader might talk about the bigger picture and articulate ultimate aspirations (Kark and Van Dijk, 2007). If such idealized messages, aimed at inspiring followers, meet with followers’ situation of survival mode and emotional exhaustion, said followers might feel uneasy with the leaders’ messages due to the discrepancy between the leader’s and followers’ emotional level (Damen et al., 2008). While leaders’ emotional level might be intense and positive, followers’ emotional level in such a prevention survival mode would be of low intensity and high negativity (Feldman Barrett and Russell, 1999). We expect that such a clash might harm followers’ experience of purpose and significance. First, while the purpose of followers in such a state is to solve the immediate, concrete, short-term problems, leaders’ purpose would be abstract and long-term (Kark and Van Dijk, 2007). If followers cannot see any connection between leaders’ higher-order abstract meaning provision and the ways they themselves construe their immediate situation, their sense of purpose of their current work might be distorted (Carton, 2017). Second, whereas the follower in such a state might consider solving the immediate problems as worthwhile and significant, the leader may undermine followers’ feelings of significance by referring to an abstract, idealistic, and positive future. Leaders, who overlook the precarious and stressful situation of their followers and focus on ultimate, higher-order, abstract aspirations, are likely to harm followers’ emotional and evaluative baseline. Overall, leaders’ ignorance of followers’ emotional situation and survival mode, is likely to interact with their meaning making process and reduce followers’ sense of meaning at work.

Proposition 5: Followers’ characteristics (misfit between followers’ and leaders’ values as well as followers’ emotional exhaustion) will moderate the effect of leaders’ meaning making attempts on followers’ sense of work meaningfulness, such that (a) followers’ experience of a misfit between their own and leaders’ values, or (b) followers’ emotional exhaustion, will reduce this relationship.

### Job Design: Irrelevant and Disconnected

Another way through which leaders influence followers’ meaning is through structuring the work of their followers and hence, through job design (Hackman and Oldham, 1980). This mode of influence has also been referred to as indirect leadership (Kerr and Jermier, 1978). Amabile and Kramer (2011) describe three ways in which managers unwittingly drain work of its meaning through job design. First, managers may destroy employees’ sense of ownership of their work, for example, by frequent and abrupt reassignments. Second, managers may convey the message that the work employees are doing will never see the light of the day, for example, by constantly changing their priorities or their minds. Third, leaders may forget or neglect to inform employees about unexpected changes in a customer’s priorities so that followers’ work efforts lose their relevance. All three ways refer to some form of disconnection between followers’ work, their self, and their ability to make a meaningful contribution (Amabile and Kramer, 2011; Bailey and Madden, 2016). The frequent and abrupt reassignments might hurt followers’ sense of meaning most if leaders do not provide a logical reason for changing followers’ work activities. Consequently, followers’ sense of coherence might be distorted because followers might struggle to understand the logic behind leaders’ decision and to see how their piece of work fits into the bigger picture (Heintzelman and King, 2014). Beyond that, the lack of comprehensibility and coherence is likely to harm followers’ sense that their work has significance and serves a purpose (Martela and Steger, 2016). Furthermore, leaders who structure or delegate tasks so that they never can be finished, make it difficult for followers to derive meaning from work. Even if the followers’ assignments bear inherent significance and value to them, if followers feel their work will “never see the light of day” then effectively, their work is futile and meaningless. Without a final product or any sense that the process has reached an endpoint, there are far less opportunities for the follower to receive positive feedback on their work, which could reinforce its worth (Grant, 2007). If leaders, customers, or any other beneficiaries, that followers’ work is supposed to serve, change their priorities, followers’ work loses its relevance. In such cases, followers are likely to become frustrated and upset by the loss of their work significance (Chadi et al., 2017).

The aforementioned negative effects might even be exacerbated when leaders try to inspire followers with some higher-order goal, while simultaneously giving them ever changing, non-achievable tasks. Consider a leader who paints a picture of an idealized future and of how the organization will make a difference in the world, but at the same time randomly re-assigns tasks or gives irrelevant tasks (McGregor and Little, 1998). Followers might become very frustrated, as there is great dissonance between what they do and can achieve in reality and what they are being inspired to achieve and contribute to (Carton, 2017). Their aspiration might become very distant, unreachable, and even ridiculous through the huge experienced contrast between reality (which is full of irrelevant, mismanaged tasks) and their aspirations, such as changing the world (Schwarz and Bless, 1992). In addition, the lack of consistency between the goals set by leaders and their actions might cause followers to question the goals themselves as they grow increasingly skeptical of their leaders’ judgment due to their poor business running and work structuring capabilities. The inconsistency experienced by followers might cause them to question why they are doing their work or why they are there (Pratt and Ashforth, 2003), signaling that they are uncertain about the purpose and significance of their work and their contribution.

Disconnecting followers from products and results is not the only form of disconnection a leader can bring about to impair followers’ sense of meaningfulness. Sometimes leaders create interpersonal isolation or distance among their followers or between themselves and their followers. Both create disconnection from supportive relationships, which might harm followers’ work meaningfulness (Rosso et al., 2010; Bailey and Madden, 2016). Research on relational job design has shown that interactions with others at work, such as leaders, coworkers, or customers helps to experience the impact and purpose of one’s work (Grant, 2007). However, if leaders create an isolating work environment, in which there is little to no interpersonal interaction with others or in which the leader does not engage in supporting behaviors, followers might experience feelings of isolation and loneliness. Such a lack of interpersonal connectedness might thereby reduce followers’ self and collective efficacy beliefs, due to decreased levels of social identification (Kark et al., 2003). If leaders talk about a high-level aspiration while neglecting to provide some type of support or additional resources to followers, followers’ sense of meaning might be reduced by the felt impossibility to take a meaningful step toward the higher goal. Leaders’ espoused high-level aspiration might oppress followers, when they feel isolated and receive limited or no social support.

Proposition 6: Job design aspects will moderate the effect of leaders’ meaning making on followers’ sense of meaning, such that followers’ sense of meaning will be reduced, when the job is designed as irrelevant and disconnected from others.

### Follower-Related Consequences of Reduced Work Meaningfulness

We argue that the decrease of followers’ work meaningfulness will have negative consequences for followers’ cognitions, well-being, and behaviors. In particular, we expect that followers, whose sense of meaningfulness has been reduced, will show a heightened level of cynicism. Cynicism has been defined as both a general and specific attitude, characterized by frustration, hopelessness, and disillusionment, as well as contempt toward and distrust of a person, group, ideology, social convention, or institution (Andersson, 1996). In the present case, we expect that followers will experience frustration, hopelessness, and disillusionment because their high expectations of realizing their self through work might have been disenabled or prevented by their leaders.

We also expect that a lower level of work meaningfulness will lead to a lower level of well-being, including heightened levels of negative affect, distress, exhaustion and depression among followers and reduced levels of vitality and thriving (Sonnentag, 2015). While prior research has shown that helping others at work or through one’s work increases one’s positive affect (e.g., Sonnentag and Grant, 2012), we expect that a low level of meaningfulness at work increases one’s negative affect and lowers followers’ overall well-being based on the frustration of both meaning pathways (lack of realization and justification). On the one hand, followers’ limited possibilities to realize their self and their potential at work is likely to lead to negative affect, distress, and exhaustion, which might potentially culminate into depression or sickness. Followers are likely to be disappointed and frustrated, when they gain awareness of the gap between their actual self at work, which performs activities with low impact on others or with low personal significance, and their ideal self at work, which would express their true self or grow personally (Higgins, 1987; Carver and Scheier, 1990). On the other hand, followers’ lack of meaningful accounts to explain and justify the value of their work might reduce their well-being and their level of experienced vitality and thriving. Followers, who question why their work is meaningful for themselves and/or for others, and thus, cannot competently justify the worth of their work (Lepisto and Pratt, 2017), might feel useless, distressed, irritated, or depressed. Hence, it is quite likely that these followers score lower on well-being.

Moreover, we expect that followers’ reduced work meaningfulness influences followers’ behaviors; in particular, we expect a higher level of disengagement. Disengagement is defined as the uncoupling of selves from their work roles, which means that followers are physically uninvolved in tasks, and that role demands guide their task behavior (Kahn, 1990). When followers’ sense of meaning at work is reduced, meaning they consider their work as less personally significant and worthwhile, it is likely that such followers reduce their efforts to fulfill their job and work activities.

Proposition 7: Followers’ reduced sense of meaningfulness will (a) enhance followers’ cynicism, (b) reduce followers’ well-being, and (c) enhance followers’ disengagement.

### Organizational-Level Consequences of Reduced Work Meaningfulness

Furthermore, we suggest that followers’ decreased levels of work meaningfulness will have organizational-level consequences. First, we argue that the level of corrosive organizational energy increases when followers’ work meaningfulness decreases. Corrosive organizational energy describes the level of shared destructive energy within the organization characterized by aggression and destructive behavior (Bruch and Ghoshal, 2003). Importantly, corrosive energy entails self-reinforcing negativity (Bruch and Ghoshal, 2003), which means that if some followers are frustrated by their work and/or their leader, these followers are likely to infect other organizational members with their negative feelings, thoughts, and behaviors. It is likely that the experience of frustration and cynicism of followers not only results in disengagement but also evokes destructive behaviors against the organization creating a toxic, destructive, and corrosive work environment.

Additionally, based on previous insights, we expect that reduced levels of work meaningfulness leads to higher turnover rates in organizations. For example, Lancaster and Stillman (2010) cited the absence of meaningfulness as a key reason for turnover, and the meta-analysis of Humphrey et al. (2007) on work design features provided evidence that there is a negative linkage between experienced work meaningfulness and turnover intentions. We argue that if organizations and leaders cannot meet followers’ desire for meaningful work but rather reduce followers’ feelings of meaning at work, followers are very likely to leave the organization.

Moreover, we expect that a decrease in followers’ work meaningfulness leads to lower organizational productivity. If followers do not fully engage in their tasks, they are less productive, which is likely to yield an overall lower level of organizational productivity (Harter et al., 2002). In addition, due to followers’ lowered meaning coherence, which implies that followers have only a fuzzy shared mental understanding of the overall purpose of their work (Carton et al., 2014), their forms of cooperation and team collaborative work might be less effective, so that organizational productivity is reduced.

Proposition 8: Followers’ reduced sense of meaningfulness will (a) increase corrosive organizational energy, (b) increase turnover rates, and (c) decrease organizational productivity.

## Discussion

According to Shamir et al. (1993) identity motivational theory of leadership, followers’ sense of meaning and their need to find meaning is a significant aspect of their organizational life. Our conceptual model brings forth a set of different conditions under which leaders’ meaning making can have detrimental effects on followers’ work meaningfulness. We suggest different aspects that can interact with leaders’ provision of a sense of meaning and lead to reduced meaning and eventually bring negative consequences for followers and organizations.

The theory of positive organizational psychology highlights the role of positive upward emotional spirals in organizations (Fredrickson and Joiner, 2002) as a power that feeds growth and advancement. Scholars in this field also highlight the importance of relationships and interactions that are “life giving” (Dutton and Heaphy, 2003; Kark, 2012). However, leaders’ and leader-follower dynamics can actually lead to negative downward spirals and to interactions that are “life depleting,” when leaders harm followers’ sense of meaningfulness. This spiral can become contagious and lead to further negative effects in the organizational life.

Harming one’s sense of meaningfulness may be easier and more common than enhancing followers’ sense of meaningfulness. This is due to a negativity bias that is evident in numerous psychological phenomena (Baumeister et al., 2001). These works show that events with a negative valence (e.g., losing capital, breaking up with a friend, and being criticized) will have a stronger and longer lasting influence on people than similar events that have a positive valence (e.g., gaining capital, making friends, and receiving positive feedback). This was named the *negativity bias* (Rozin and Royzman, 2001) and the *asymmetry effect* (Peeters, 2002). With regards to emotions and possibly work meaningfulness, the effects of negative affect at work and within organizational life are stronger and more memorable and detailed than positive affect (e.g., Kaplan et al., 2009; van Kleef, 2009; George, 2011). This has also been demonstrated for the effect of leadership behavior (Dasborough and Ashkanasy, 2002; Sy et al., 2005; Kark et al., 2017). For example, Kark et al. (2017) found that it is easier for leaders to hinder creative behaviors of followers than to encourage such behaviors, since people are more attentive to negative versus positive leadership behaviors and signals to the prevention versus the promotion self-regulatory focus. When leaders are monitoring and looking for mistakes and exceptions, and when they elicit a self-regulatory focus of prevention, this may have a stronger effect on hindering creativity than the effect of charismatic and transformational leadership to promote novel ideas, thinking on the ideals and, creativity. This phenomenon was acknowledged by Amabile (1998, p. 77). In her words: “When I consider all the organizations I have studied and worked with over the past 22 years, there can be no doubt: creativity gets killed much more often than it gets supported.”

With regards to work meaningfulness, this implies that the model we suggest, which aims to understand how contextual characteristics in the leader–follower process may harm followers’ meaningfulness, may have significant effects on the ability of managers to maintain followers’ meaningfulness without harming it. Furthermore, we contribute to the leadership and followership literature that considers the ‘dark side of leadership’ by showing under which conditions leaders might have a negative influence on followers’ motivating forces, namely meaningfulness.

By incorporating the recent advancements of research on work meaningfulness (Martela and Steger, 2016; Lepisto and Pratt, 2017), we developed a more detailed understanding regarding the particular dimensions of work meaningfulness, i.e., coherence, purpose, and significance, and the underlying pathways, i.e., the realization or justification perspective, that can be harmed by the interaction of leaders’ meaning making and the identified conditions. At times when many employees search for a deeper meaning at work (Cascio, 2003; Twenge, 2006), leaders might be asked and encouraged to offer new solid accounts for the worth of employees’ work. However, our model indicates that the outcome of this endeavor hinges upon many different facets including the ways followers perceive and interpret leaders’ attempts and to which degree leaders manage to reach an alignment between their, the organization’s, and followers’ values. While recent research on respectful inquiry, a leadership technique of asking followers open questions and attentively listening to them (Van Quaquebeke and Felps, 2018), suggests that leaders might be able to incorporate followers’ perspective by engaging in listening, leaders still need to provide their point of view, show their value base and take a stance, when providing accounts for the meaningfulness of work.

Moreover, our research extends prior writings on the critical perspective on the management of meaning (Lips-Wiersma and Morris, 2009; Bailey et al., 2017) by revealing the factors under which leaders’ meaning making might harm followers’ work meaningfulness. Although the answers to the question of whose responsibility meaningful work is (Michaelson, 2011) might vary, our research is in alignment with the scholars of the critical perspective of meaning management inasmuch as both would respond to the question that it is the responsibility of leaders *not* to dismantle followers’ work meaningfulness.

### Research Agenda

Our conceptual model offers a wide terrain for future research. It suggests that there are multiple contextual conditions that interact with the process of meaning making to effect its outcomes. We offer various dimensions for underrating the process of work meaningfulness hindrance as a multi-focal process. In order to study this complex phenomenon, qualitative studies would be helpful at a first stage. In-depth interviews and ethnographies that explore the complex processes of meaningfulness hindrance over time will enable a more nuanced and deeper understanding of the mechanisms of this process. At a later stage, quantitative studies are warranted. Following and extending the work of Martela and Steger (2016) and Lepisto and Pratt (2017), our model proposes that leaders may harm followers’ work meaningfulness by reducing their sense of coherence, purpose, and significance. This three-dimensional model of work meaningfulness needs the development of a new scale that can be used to explore how leaders contribute to followers’ sense of meaningfulness. Thus, in future studies researchers can explore the ways in which the different aspects of reducing meaning affects these different components of the meaning making process. The meaningfulness scale can be used to explore the negative side looking into what extent leaders can harm meaningfulness. To be even more concrete, future studies might rely on experience sampling designs. Followers might report their level of perceived work meaningfulness multiple times throughout a working day, while they might also report the level of perceived meaning making of leaders in combination with the attributed intentionality on leaders’ behaviors (Proposition 4). Last, laboratory experiments may be used to evaluate in a better controlled environment the causality of leaders’ behavior and contextual aspects and how they influence the meaning reduction process. For example, future laboratory studies might build upon existing experimental studies on meaningless tasks (Ariely et al., 2008; Chadi et al., 2017) and create jobs and scenarios in which leaders’ meaning making attempts are varied in combination, for example, with the job design (Proposition 6) or with the followers’ current emotional state (e.g., survival mode; Proposition 5).

Future research might also develop further conditions, which might harm followers’ work meaningfulness. While we focused on leaders, followers, their relationship and job design, considering these the major building blocks of the factors harming followers’ work meaningfulness, future studies might add factors to each of our categories or even suggest new categories. For example, the phenomenon of multiple team memberships (O’Leary et al., 2011) and the accompanying constant change of team constellations might imply that followers lose track of a coherent understanding of what their work is contributing to. Regarding the consequences of reduced levels of followers’ work meaningfulness, future research might also investigate which implications the lowered meaningfulness has for followers’ level of intrinsic motivation and behavioral coping strategies. We suspect that certain individuals might react with a general reduction of their (intrinsic) motivation, while others might engage in certain behaviors to cope with or change the situation, for example, engage in job crafting. Revealing the personal (e.g., proactive personality, socio-economic status) and contextual factors (e.g., organizational climate), which might evoke certain reactions, represents an exciting avenue for future studies. Overall, we hope that our broad framework will inspire more focused and deeper theoretical developments on different aspects of the presented model, as well as empirical studies that focus on some of our more specific propositions.

### Limitations

Although the current model offers a wide variety of contextual conditions that are likely to harm followers’ sense of work meaningfulness, we could not come up with an exhaustive list of conditions that harm followers’ work meaningfulness. Future studies may want to consider additional aspects of the leader-follower relationship, such as gender bias or followers’ attachment orientation, leaders’ abusive supervision, the financial climate, time pressure of the leaders and other aspects that may harm the meaningfulness making process. Importantly, while our model mainly considered particularly negative factors, such as leaders’ dark triad traits, also seemingly positive characteristics and circumstances might undermine followers’ sense of work meaningfulness. Furthermore, in our conceptual model each contextual condition is offered as a stand-alone variable. However, in the organizational life, these different aspects interact. For example, leaders who are perceived as Machiavelli may also be seen as unethical and may build a relationship with employees that is characterized by a low level of trust. Thus, future work needs to further develop the interaction among different contextual aspects, or conditions in which more than one of these contextual characteristics is evident, in order to obtain a more complex theoretical perspective. Moreover, our model is focused on the individual level. However, employees in organizations work in teams and in workgroups and their sense of meaningfulness is likely to be shaped and effected by other team members, the team environment and the heterogeneous specific relationships that different team members construct with the leader. Last, our model explores one direction of influence in which leaders shape and harm followers’ sense of meaningfulness. However, followers are also active agents and they may also harm leaders’ sense of work meaningfulness. Thus, future studies should also explore the opposite direction of influence or multiple and reciprocal directions of influence on reducing meaningfulness.

It is worthy to note that in most circumstances managers may be attuned to fostering work meaningfulness and may be conscious of their behaviors aimed to elicit and affect the sense of meaningfulness. However, the behaviors and conditions that reduce the sense of meaning may be more hidden and managers may act upon them without full awareness. This dynamic should be explored in future studies.

### Practical Implications

Our research makes a major step forward in better understanding the erosion of meaningful work through leaders. While an answer to the quest for meaningful work could be to increase the meaning making of leaders, our research shows that such behavior could decrease followers’ work meaningfulness. Thus, our conceptual work suggests that leaders should be trained and taught in the ways they should refrain from harming employees’ sense of meaningfulness. Under certain conditions of personality characteristics such as the dark triad, organizations should be attuned to better select managers that are low on such behaviors and personality structure, in order to reduce the possible negative effects of such leaders on employees’ sense of meaningfulness. Furthermore, if there is a negativity bias, and an asymmetrical effect of fostering versus hindering a sense of meaningfulness, leaders should be aware of their ability to harm more than to construct a sense of meaning. This implies that managers, practitioners and HR personnel should be mindful of followers’ sensitivity to this negative dynamic, and attempt to refrain from giving rise to such a dynamic that can result in the loss of followers inner meaningful ‘songs.’

## Author Contributions

This project was a joint effort. Both authors developed the theory and ideas and contributed to writing the manuscript. The manuscript was written by both PK and RK, however, PK took the leading role in the writing process.

## Conflict of Interest Statement

The authors declare that the research was conducted in the absence of any commercial or financial relationships that could be construed as a potential conflict of interest.
